# Performance of Cloud-Hosted Large Vision-Language Models on the Japanese National Examination for Clinical Laboratory Technicians: Comparative Benchmarking Study

**DOI:** 10.2196/84266

**Published:** 2026-09-11

**Authors:** Kota Murakami, Ryosuke Matsuzawa, Yuya Tahara-Arai, Haruka Ozaki

**Affiliations:** 1 Master’s Program in Medical Sciences, Graduate School of Comprehensive Human Sciences, University of Tsukuba, Ibaraki, Japan; 2 Bioinformatics Laboratory, Institute of Medicine, University of Tsukuba, Ibaraki, Japan; 3 Laboratory for AI Biology, RIKEN Center for Biosystems Dynamics Research, 6-7-1 Minatojima Minamimachi, Chuo-ku, Kobe, Hyogo, 650-0047, Japan, 81 78-306-0111; 4 Doctoral Program in Medical Sciences, Graduate School of Comprehensive Human Sciences, University of Tsukuba, Ibaraki, Japan; 5 Ph.D. Program in Humanics, School of Integrative and Global Majors, University of Tsukuba, Ibaraki, Japan

**Keywords:** large vision-language models, Japanese National Examination for Clinical Laboratory Technicians, multimodal reasoning, medical AI, model performance evaluation

## Abstract

**Background:**

Cloud-hosted large vision-language models (LVLMs) often outperform open-weight models on multimodal benchmarks, but their applicability to health care examinations that require both text and image reasoning remains unclear. Existing studies on Japan’s National Examination for Clinical Laboratory Technicians mainly used text-only settings and a limited set of models.

**Objective:**

This study aimed to evaluate the accuracy and applicability of cloud-hosted LVLMs on this examination, quantify the contribution of images, examine alignment with human performance across medical subfields, and test generalizability on questions administered after the models’ knowledge cutoff dates. We aim to provide a standardized benchmark of baseline multimodal performance under deployment-realistic conditions without additional fine-tuning.

**Methods:**

For this comparative benchmarking study, we built the Kensagishi question-answer dataset (Kensagishi QA), a benchmark of 800 questions from the examination (2020‐2023), including text-only and image-based questions, in a standardized structured JSON format, and strict scoring criteria (all correct options required). We tested GPT-5, GPT-4o, GPT-4o-mini, Gemini 1.5 Pro, Gemini 2.5 Pro, and Neva-22B using a uniform “numbers-only” response prompt; images were provided as Base64-encoded data when available. Accuracy was computed overall, by item type, and by medical subfield. Human reference was drawn from published examination results. Spearman correlation assessed model-human alignment. Generalization was evaluated on 200 questions from the 71st examination (2025), released after the models’ knowledge cutoffs.

**Results:**

Top-performing models exceeded the 60% passing threshold. GPT-5 achieved 93.9% (95% CI 92.0%‐95.4%) accuracy with images and 88.2% (95% CI 85.7%‐90.2%) without images, Gemini 2.5 Pro 92.5% (95% CI 90.5%‐94.1%) and 86.8% (95% CI 84.2%‐88.9%), and GPT-4o 81.7% (95% CI 78.8%‐84.2%) and 79.8% (95% CI 76.8%‐82.4%), respectively. Providing images markedly improved accuracy for GPT-5, Gemini 2.5 Pro, and GPT-4o, whereas Neva-22B showed no improvement. Model-human rank correlations by medical subfield were moderate for GPT-4o (Spearman ρ=0.61; 95% CI –0.03 to 0.90; *P*=.06 with images) and statistically significant for Gemini 1.5 Pro without images (ρ=0.67; 95% CI 0.07‐0.91; *P*=.03). On 200 postknowledge cutoff questions from the 71st examination (2025), model accuracies were comparable to historical averages (eg, GPT-5 91.5%, 95% CI 86.8%‐94.6%; Gemini 2.5 Pro 90.7%, 95% CI 85.8%‐94.0%; GPT-4o 82.0%, 95% CI 76.1%‐86.7%), indicating no material performance degradation on unseen questions.

**Conclusions:**

Cloud-hosted LVLMs show strong performance on this examination, and visual input is a key driver of accuracy for leading models. Models that better mirror human difficulty patterns (eg, GPT-4o) offer complementary insights for educational analytics, whereas the highest-accuracy models (eg, GPT-5 and Gemini 2.5 Pro) may be preferable when correctness is paramount. To our knowledge, this is the first systematic, multimodel evaluation isolating visual input that extends prior text-only, few-model studies and provides Kensagishi QA to guide educational deployment of medical AI.

## Introduction

Large language models (LLMs) are a type of natural language processing technology that acquire the ability to understand and generate human language by learning from massive amounts of textual data. These models have demonstrated high performance across a wide range of tasks, including question answering, summarization, translation, and reasoning, with rapidly expanding applications [1-4]. In recent years, large vision-language models (LVLMs), which are capable of handling not only text but also multimodal information including images, have also emerged. These models are designed to understand and generate both visual and textual information simultaneously, enabling their application to a broader range of tasks such as image-based question answering, image captioning, and visual reasoning [2,4]. Notably, recent multimodal benchmarks (eg, VisionArena [5] and MMStar [6]) indicate that proprietary, cloud-hosted, closed-weight LVLMs currently outperform open-weight, self-hosted models.

The rapid advancement of LLMs has also extended into the medical domain, where evaluations of performance on the United States Medical Licensing Examination (USMLE) have become increasingly common. Prior studies using benchmark datasets such as MedQA, which contains USMLE-style questions, have demonstrated that LLMs can answer questions requiring domain-specific knowledge [7]. For instance, Med-PaLM 2, developed by Google, achieved an accuracy of 86.5% on the MedQA dataset and received favorable evaluations compared with human physicians’ responses [8]. In addition, the LLM-MedQA system using the Llama3.1:70B model reported approximately 7% improvements in accuracy and *F*_1_-scores under zero-shot settings by incorporating a multiagent architecture and similar case-generation techniques [9]. Furthermore, with the integration of Semantic Clinical AI (SCAI), which embeds structured clinical knowledge into LLMs, performance has significantly improved, achieving up to 95.1% accuracy on USMLE Step 3, surpassing conventional models by a considerable margin [10]. In a similar effort in the Japanese context, a benchmark dataset named IgakuQA, based on questions from the Japanese National Medical Licensing Examination, was developed to explore the applicability of LLMs while accounting for linguistic and health care system differences [11]. Moreover, LLMs have also been evaluated in supporting the creation of multiple-choice questions for the Japanese National Nursing Examination, particularly in generating and assessing distractors [12].

In contrast, although text-only LLMs have been actively evaluated on medical licensing examinations, the applicability of LVLMs to health care–related national examinations—particularly those requiring both textual and visual reasoning—remains underexplored. For the Japanese National Examination for Clinical Laboratory Technicians, existing reports have primarily assessed a limited number of general-purpose LLMs, such as ChatGPT-3.5 and GPT-4 [13-15], often in text-only settings. As a result, it is still unclear how modern, cloud-hosted (closed-weight) LVLMs perform on this examination and to what extent visual input contributes to accuracy.

This study identifies 2 primary gaps. First, there is no publicly available, well-structured dataset specifically tailored to the National Examination for Clinical Laboratory Technicians (including paired image assets and metadata). Consequently, considerable effort is required to conduct reproducible cross-model comparisons or assess newly developed systems. Second, prior work has not provided a systematic, multimodel evaluation of LVLMs that isolates the contribution of images; although some attempts have included image-dependent items (eg, peripheral blood smear morphology classification and electrophoresis band pattern interpretation), coverage has been limited and insufficient for comprehensive multimodal comparison. Taken together, these gaps highlight the need for a standardized, reproducible benchmark that enables fair cross-model comparison under controlled conditions.

Given this background, the aim of the present study was to conduct a multifaceted evaluation of the applicability of cloud-hosted LVLMs to the medical domain, using the National Examination for Clinical Laboratory Technicians as a case study. In particular, we focused on the impact of visual information, in addition to textual information, on model accuracy. To this end, we constructed a custom benchmark dataset, Kensagishi question-answer (Kensagishi QA), based on past questions from the Japanese National Examination for Clinical Laboratory Technicians. By systematically comparing the performance of multiple cloud-hosted LVLMs, we sought to elucidate their strengths and limitations and to provide fundamental insights to support future advancements in medical AI.

## Methods

### Construction of a Dataset Based on the Japanese National Examination for Clinical Laboratory Technicians

This study was designed as a comparative benchmarking study to develop an examination benchmark and evaluate the performance of multiple cloud-hosted LVLMs. To support this evaluation, we constructed a novel benchmark dataset, Kensagishi QA, comprising questions from the 66th to 69th National Examination for Clinical Laboratory Technicians conducted between 2020 and 2023. The official examination questions for each year are publicly available in PDF format [16-19]. These materials were obtained from the official website of the Ministry of Health, Labour and Welfare (MHLW), where the content is provided under the Public Data License (version 1.0; PDL1.0), unless otherwise noted. Redistribution of the structured question data in our GitHub repository complied with the applicable license terms, including appropriate attribution.

**Figure 1.**
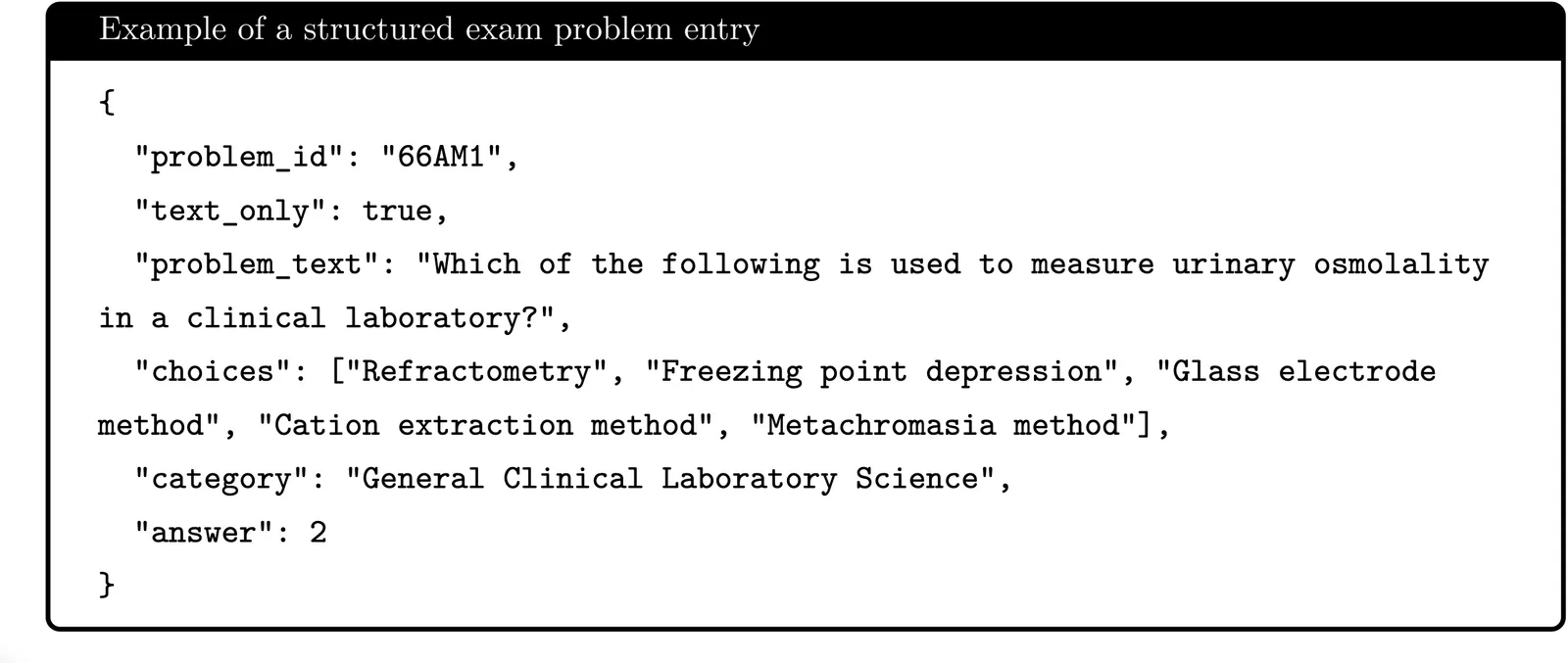
Example of a structured dataset entry from the Japanese National Examination for Clinical Laboratory Technicians. The question and choices were translated from the original Japanese text for illustrative purposes.

From these PDF files, we extracted the question and answer choices and converted them into a machine-readable JSON format (Figure 1). During the conversion process, the data were structured to include fields such as question ID, question text, answer choices (1-5), correct answer, and question type (text/image).

The National Examination for Clinical Laboratory Technicians includes a mix of text-only questions (hereafter referred to as text questions) and questions containing medical images or diagrams (hereafter referred to as image questions). In the constructed dataset, these questions were categorized by type, allowing for separate performance evaluations of each question format.

All questions follow a 5-choice format and consist of either single-answer or multiple-answer types, where a single question may have more than one correct option. Each year, 200 questions are administered—100 in the morning and 100 in the afternoon—resulting in a total of 800 questions collected from consecutive examination years (2020‐2023). Of the 800 questions, 670 (83.75%) were classified as text questions, whereas 130 (16.25%) were identified as image questions. The image questions include prompts that require interpretation of visual information, such as blood smears, histopathological images, device output screens, and tabular data, thereby necessitating visual input processing.

### Evaluation of Accuracy of LVLMs on the Japanese National Examination for Clinical Laboratory Technicians

In this study, we evaluated the accuracy of multiple LVLMs on questions from the National Examination for Clinical Laboratory Technicians. To specifically assess performance on questions involving images, we selected LVLMs capable of processing both textual and visual inputs.

The selection of these 6 LVLMs was intended to represent a range of model generations, providers, and practical deployment scenarios rather than to compare only the most recent state-of-the-art systems. In particular, we included multiple models released by the same provider (GPT-4o-mini, GPT-4o, and GPT-5) to examine performance differences across model generations and to assess whether earlier, lower-cost models remain viable for large-scale or educational use, in addition to evaluating the capabilities of the latest model. This reflects realistic constraints in applied settings, where the most recent model is not always adopted due to factors such as inference cost, availability, latency, and system compatibility. Furthermore, models from different providers (OpenAI, Google, and NVIDIA) were included to reduce vendor-specific bias and examine how differences in architecture and training affect multimodal performance on the same benchmark. We note that all evaluated models were accessed via cloud APIs in this study, and no local deployment or on-premises inference was performed.

An overview of the models used in this study is presented in Table 1. For Gemini 2.5 Pro, we used the preview version released on March 25, 2025 (gemini-2.5-pro-preview-03-25), prior to its general availability.

**Table 1.** Comparison of providers, API names, parameter sizes, release dates, and knowledge cutoffs for each large vision-language model.

Model	Provider	API name	Number of parameters	Release date	Knowledge cutoff
GPT-4o-mini	OpenAI	gpt-4o-mini-2024-07-18	Not disclosed	July 18, 2024	October 2023
GPT-4o	OpenAI	gpt-4o-2024-08-06	Not disclosed	August 6, 2024	October 2023
Gemini 1.5 Pro	Google	gemini-1.5-pro	Not disclosed	September 24, 2024	May 2024
Gemini 2.5 Pro (Preview)	Google	gemini-2.5-pro-preview-03-25	Not disclosed	March 25, 2025 (Preview)	January 2025
Neva-22B	NVIDIA	nvidia/neva-22b	22 billion	Not disclosed	Not disclosed
GPT-5	OpenAI	gpt-5-2025-08-07	Not disclosed	August 7, 2025	September 30, 2024

The National Examination for Clinical Laboratory Technicians includes multiple-answer questions in which more than one option may be correct. To accommodate this format, we adopted a strict evaluation criterion: a response was considered correct only if all correct answer choices were selected. Partial matches were regarded as incorrect.

In addition, some examination questions include images but can still be answered correctly based solely on the answer choices or surrounding textual context. However, from the perspective of evaluating AI model performance, it is essential to assess the impact of image presence on accuracy appropriately. Therefore, we clearly distinguished between text-based and image-based questions and calculated the accuracy for each category separately.

For image questions, the image file corresponding to each question ID was encoded in Base64 and provided to the model along with the associated question text. This enabled multimodal input handling for questions that include visual content. Instructions to each model were given based on a standardized prompt format. Specifically, the models were presented with the question text and answer choices, followed by an explicit instruction to “respond with only the number(s) of the correct choice(s).” The prompt content is as follows:

Please provide the number(s) of the correct option(s) for the question below in the following format. If the question requires selecting only one answer, provide a single number. If it requires selecting two answers (e.g., "Choose two"), provide two numbers. Do not include any explanations or reasoning—just the number(s).

Question: {Question text}

Options: {List of options}

Please provide only the number(s) of the correct answer(s):

With this prompt format, the models were instructed to output only the option numbers (eg, “2” or “14”). The output was parsed using regular expressions (re.findall(r'\d+', text)) to extract numerical values, which were interpreted as the selected choice numbers. This approach was designed to robustly handle variations in the response format, such as “1 4” or “1,4.”

For all questions, each model received the question text and answer choices as textual input, and for image questions, the corresponding visual data were also provided. The images were encoded in Base64 format and provided alongside the text.

The images used in this study were screenshots extracted from the original examination materials. No image preprocessing such as resizing, cropping, or normalization was applied. Each image was used in its original resolution and directly encoded into Base64 format before being provided to the models.

The model outputs were recorded in JSONL format as pairs of question IDs and predicted choice numbers. The correctness of each prediction was determined based on the strict evaluation criterion described earlier.

Final accuracy was calculated as the proportion of correctly answered questions relative to the total number of questions. To analyze the impact of visual information on model performance, accuracy was computed separately for text questions and image questions. As a reference, we used the passing threshold for the national examination (60% accuracy) and assessed whether each model exceeded this criterion, thereby enabling comparative analysis across models.

To reduce the impact of stochastic variation inherent in the generation process, each model independently answered all questions 3 times under identical settings. The accuracy of each run was first computed, and the mean across the 3 runs was used as the model’s final accuracy. Accuracy figures display the 3 per-run values as points and 95% Wilson score CIs as error bars.

### Categorization of Questions by Medical Subfield

In this study, question categorization by medical subfield was conducted based on the official examination guidelines for the Japanese National Examination for Clinical Laboratory Technicians published by the MHLW. Specifically, the question stems and answer choices were carefully examined to interpret the medical theme and underlying intent of each question. Based on this analysis, we manually classified each question into relevant subfields, such as clinical physiology, clinical chemistry, and pathological histocytology. The categorization was performed by a single author (KM); no independent reannotation or interannotator agreement assessment was conducted, which is acknowledged as a study limitation. Using this categorization, accuracy was calculated for each subfield.

### Source of Human Accuracy Data

The human accuracy rates used in this study were based on published results from a performance analysis of the national examination [20]. Specifically, we extracted the average accuracy for each question from the previous study [20], titled “Item-by-item Accuracy Analysis,” which presents data from the 68th National Examination for Clinical Laboratory Technicians based on responses from 1896 examinees at 40 institutions.

### Correlation Analysis Between Model and Human Accuracy

To examine the correlation between model outputs and human accuracy, we calculated the Spearman rank correlation coefficient. For each medical subfield, we computed the accuracy rates for both humans and models, ranked them in descending order, and then merged the results based on the corresponding subfield names to obtain the rank difference *d_i_* for each subfield. The Spearman rank correlation coefficient, ρ*,* is defined by the following formula:


$$\rho \text{=}1\text{−}\frac{6\sum_{i}d_{i}^{2}}{n\left(n^{2}\text{−}1\right)}$$

where *d_i_* is the difference in rank between the human and model accuracies for subfield *i*, and n is the total number of subfields. This analysis quantitatively evaluates the extent to which the model’s accuracy patterns across subfields align with those observed in human performance. All *P* values are reported as exact values and formatted according to established medical journal guidelines, using an italicized capital *P* without a leading zero; values less than .001 are reported as *P*<.001. All statistical analyses were performed in Python (version 3.10.12; Python Software Foundation) using SciPy (scipy.stats, version 1.15.3; SciPy community); reported *P* values are two-sided, and statistical significance was defined as *P*<.05.

### Statistical Comparison Between Training Years and the 2025 Examination

In addition to descriptive comparisons, we conducted a statistical significance test to examine whether the declines in accuracy between the training years (2020‐2023) and the 2025 examination were statistically meaningful. For each model and image condition, we used the mean accuracy across the 3 independent runs as the final accuracy (see above) and constructed a 2×2 contingency table of correct versus incorrect responses using question-level counts (n=800 for 2020‐2023 and n=200 for 2025). Because the 3 runs are repeated measurements on the same questions rather than independent samples, the denominator was set to the number of questions rather than the number of question-run pairs. A chi-square test of independence (Pearson, without continuity correction) was then applied to each comparison.

### Specifications of the Tested LVLMs

Table 1 summarizes the specifications of the LVLMs used in this study. For each model, we document the provider, API name used, number of parameters, release date, and the final knowledge cutoff date of the training data. All inference was performed via the providers’ cloud APIs between July 2024 and October 2025; no local or on-premises graphics processing unit (GPU) compute was used, and total inference cost was modest given the limited number of API calls (approximately 800 questions×6 models×2 image conditions). Table 2 summarizes the generation settings used for each model.

**Table 2.** Generation settings used for each model in this study.

Model	Provider	Temperature	Top-p	Top-k	Number of candidates (n)
GPT-4o-mini	OpenAI	0.0	1.0	Not configurable	1
GPT-4o	OpenAI	0.0	1.0	Not configurable	1
Gemini 1.5 Pro	Google	0.0	0.95	64 (fixed)	1
Gemini 2.5 Pro (Preview)	Google	0.0	0.95	64 (fixed)	1
Neva-22B	NVIDIA	0.0	0.7	Not configurable	1
GPT-5	OpenAI	1.0	1.0	Not configurable	1

### Calculation of CIs

For every point estimate, we reported a two-sided 95% CI. For accuracy (a binomial proportion), we used the Wilson score interval [21], with the mean number of correct answers across the 3 runs as the numerator and the number of questions as the denominator (not the number of question-run pairs, because the runs were repeated measurements of the same items). The Wilson CIs reported here convey the uncertainty of each model’s accuracy estimate; interval overlap should not be interpreted as a formal between-model comparison, and no correction for multiple comparisons was applied. For the Spearman correlation between model and human subfield accuracy, we used the Fisher *z* transformation with the number of subfields as the sample size (n=10). For differences in accuracy between the 2025 and the 2020‐2023 examinations and visual-heavy versus text-heavy subfields, we used the Newcombe hybrid score method [22]. Because the benchmark enumerated all questions from the target examinations, these CIs express the precision of each estimate when the fixed question set is treated as a sample of underlying model competence rather than as sampling error from a larger population.

### Data Availability

The data used in this study are publicly available in a GitHub repository [23]. The repository contains past question data from the National Examination for Clinical Laboratory Technicians in JSONL format.

### Evaluation of Model Generalization Using Questions After the Knowledge Cutoff

To reduce the possibility that the models derived correct answers solely from memorized training data, we conducted an additional performance evaluation using questions published after each model’s knowledge cutoff. Specifically, we used all 200 questions (100 in the morning and 100 in the afternoon) from the 71st National Examination for Clinical Laboratory Technicians, administered in 2025 [24].

In this evaluation, the models were prompted using the same format as in the previous experiments. The generated responses were compared against the correct answers. As a reference, we calculated the average accuracy over the 4 earlier years (2020‐2023) and denoted this value as the “historical average.” The deviation between this historical average and the 2025 accuracy was then computed for each model. Through this evaluation, we examined each model’s ability to generalize to previously unseen questions and assessed whether the models could answer them without relying on prior exposure to the question content.

### Ethical Considerations

This study did not involve newly conducted research on human participants. We used only (1) publicly available questions from the Japanese National Examination for Clinical Laboratory Technicians and (2) aggregated item-level mean accuracy values previously published in a report [20]. The human accuracy data used in this study, namely the aggregated, item-level mean accuracy values previously published in that report, were group-level averages from 1896 examinees at 40 institutions and contained no information that could identify any individual. No individual-level data were collected or accessed, and no identifiable images are included in this manuscript or its supplementary materials. According to the Ethical Guidelines for Medical and Biological Research Involving Human Subjects, jointly issued by the Ministry of Education, Culture, Sports, Science and Technology, the MHLW, and the Ministry of Economy, Trade and Industry, the guidelines do not apply to research using only existing information that does not relate to an individual [25]. Therefore, ethics review and approval and informed consent were not required for this study.

## Results

### Overall Accuracy of LVLMs

**Figure 2.**
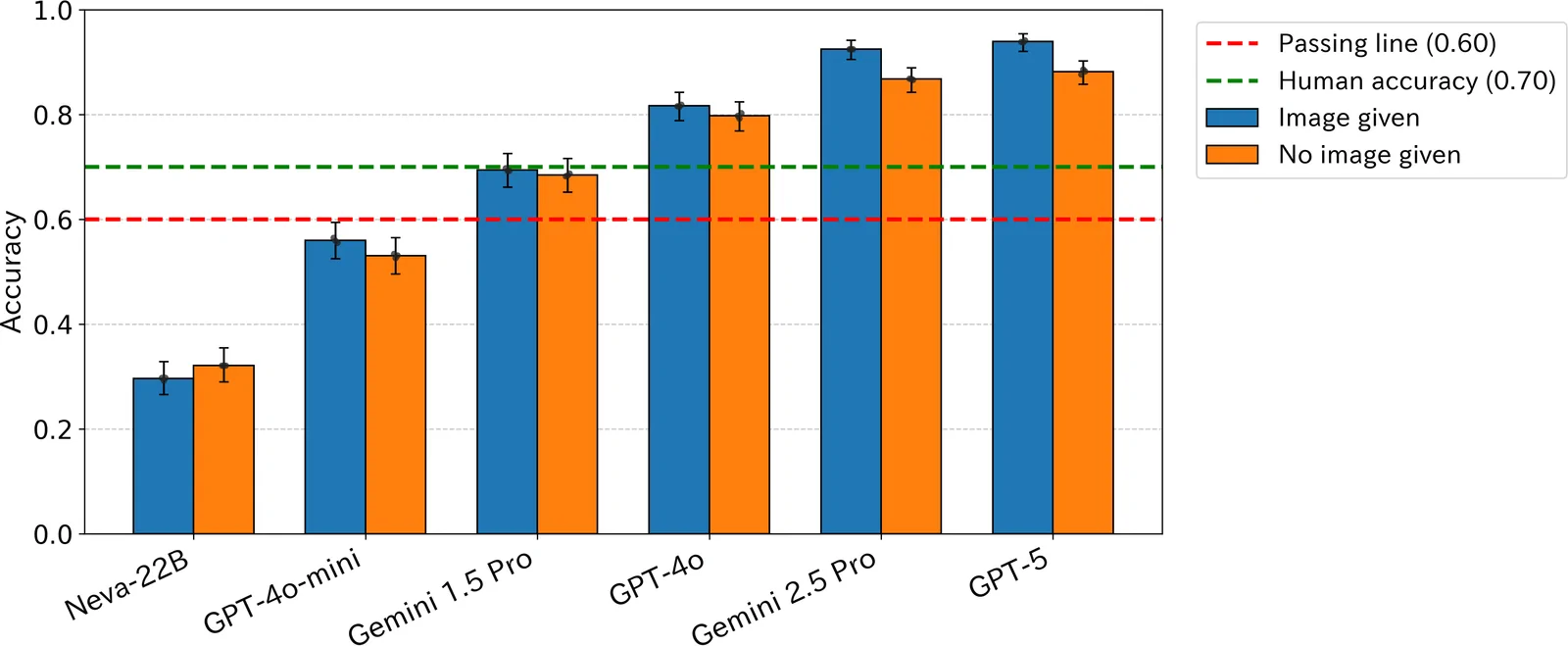
Accuracy of large vision-language models on the National Examination for Clinical Laboratory Technicians. The bar chart shows accuracies (0%‐100%) on 800 questions (text- and image-based) from the 66th to 69th examinations. The red dashed line indicates the human passing threshold of 60%, and the green dashed line represents the average human accuracy of 69.7%. Points denote the 3 per-run accuracies; error bars are 95% Wilson score CIs.

Figure 2 illustrates the accuracy achieved by each LVLM on all 800 questions from the National Examination for Clinical Laboratory Technicians. In this study, for image-based questions, we evaluated model performance under 2 conditions: an image-provided (image given) condition and a no-image (no image given) condition. For questions without associated images, identical inputs were used for both conditions. As evaluation criteria, we adopted the human passing threshold of 60% accuracy as well as the average human accuracy of 69.7%.

The overall average accuracies showed that GPT-5 achieved 93.9% with image input and 88.2% without, both substantially exceeding the passing threshold. Gemini 2.5 Pro followed with 92.5% with images and 86.8% without, also well above the benchmark. GPT-4o surpassed the threshold as well, achieving 81.7% with images and 79.8% without. Gemini 1.5 Pro recorded 69.4% with images and 68.5% without, both above the 60% benchmark.

In contrast, GPT-4o-mini scored 56.0% with image input and 53.0% without, falling below the passing threshold under both conditions. Neva-22B showed the lowest accuracy among all models, with 29.6% (with images) and 32.1% (without images). When compared with the average human accuracy (69.7%), both Gemini 2.5 Pro and GPT-4o outperformed human examinees, regardless of image availability.

### Effect of Image Input on Model Accuracy for Image-Based Questions

**Figure 3.**
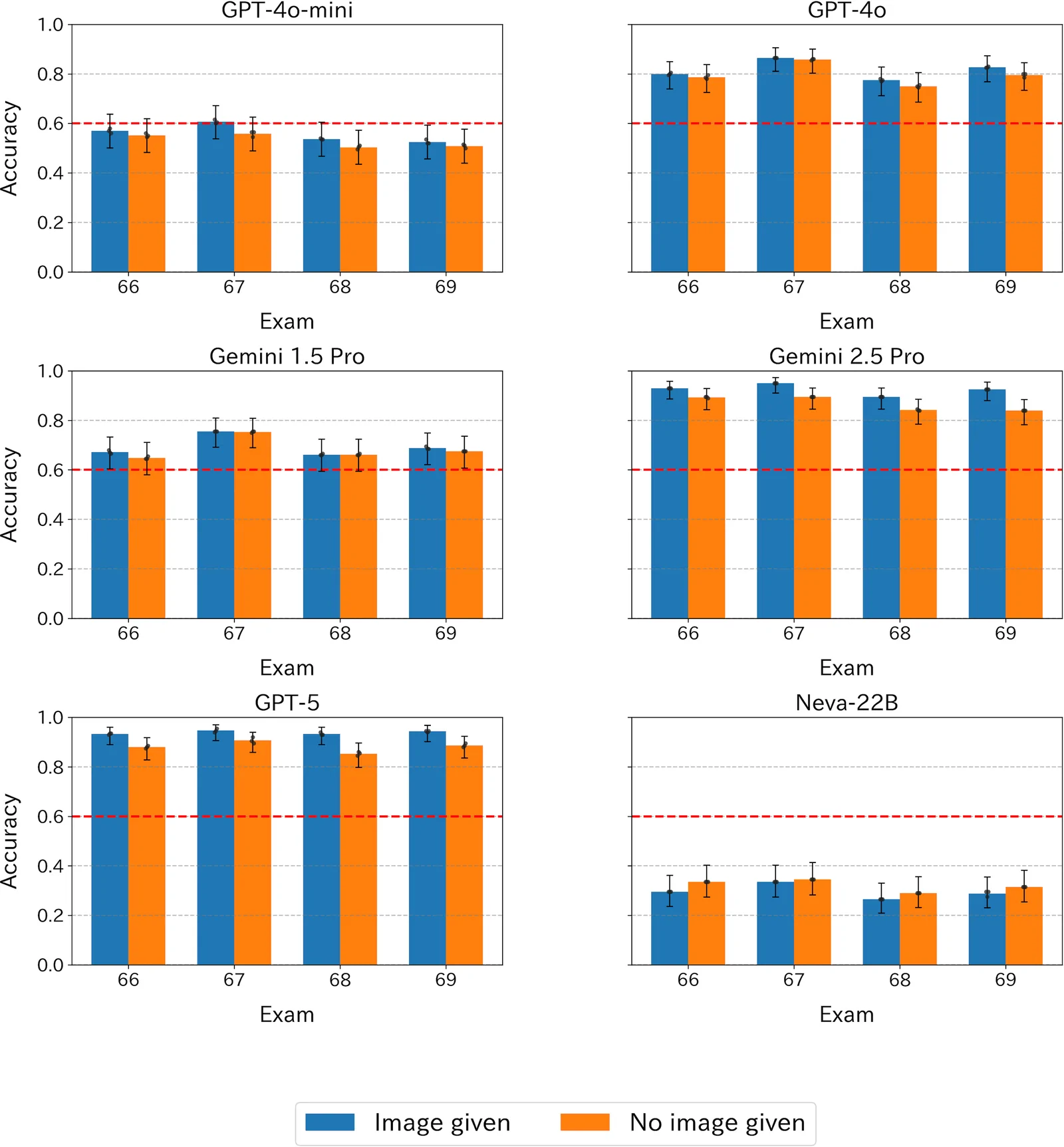
Accuracy of GPT-5, GPT-4o, GPT-4o-mini, Neva-22B, Gemini 1.5 Pro, and Gemini 2.5 Pro on all questions (text- and image-based) from the 66th to 69th National Examinations for Clinical Laboratory Technicians. The x-axis represents the examination (66th to 69th), each pooling the morning and afternoon sessions, and the y-axis represents accuracy. Blue bars indicate the condition in which images were provided for image-based questions, while orange bars indicate the condition without image input. The red dashed line marks the passing threshold of 60%. This allows comparison of the effect of image input on overall accuracy across models and examination sessions. Points denote the 3 per-run accuracies; error bars are 95% Wilson score CIs.

**Figure 4.**
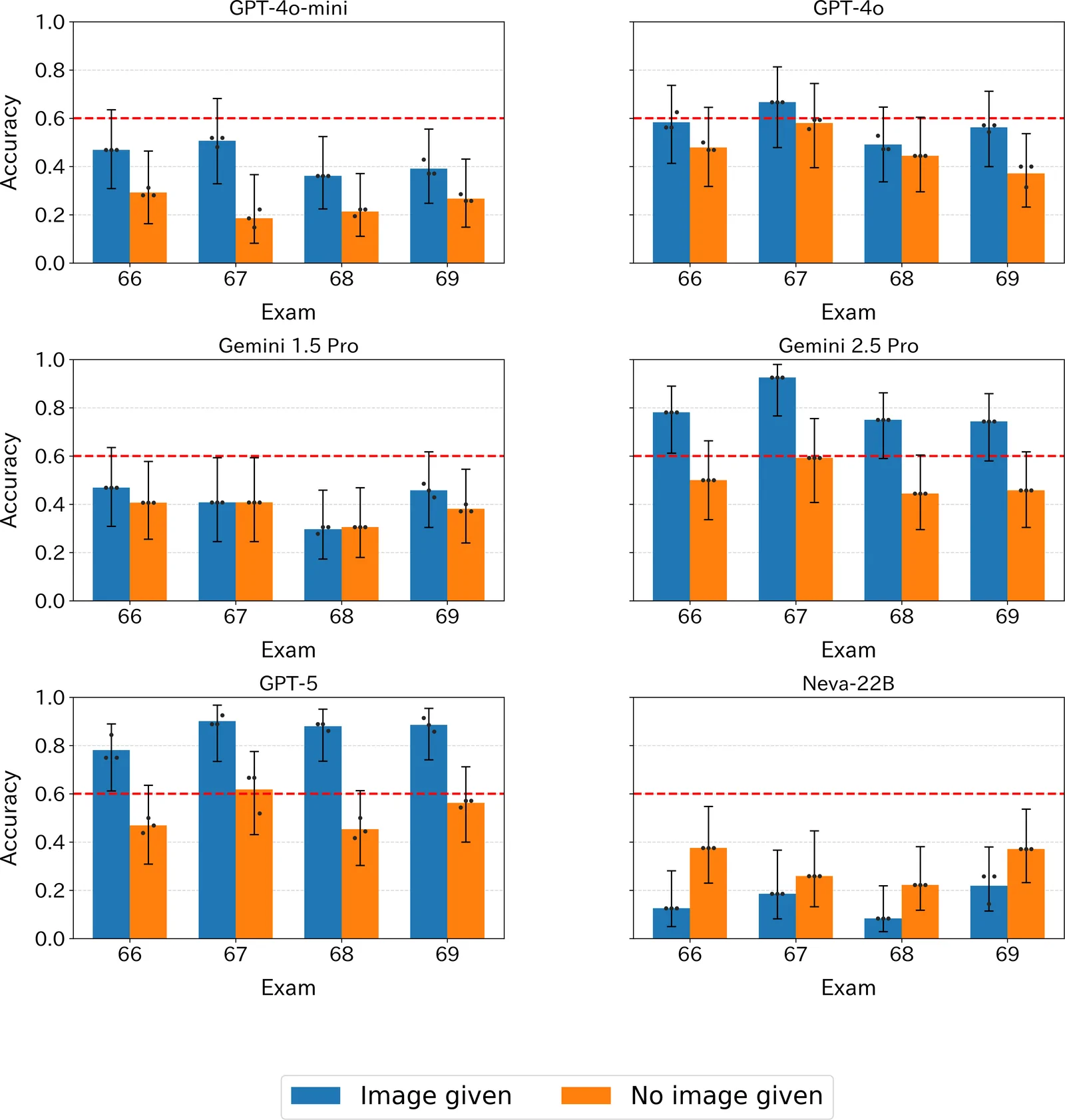
Accuracy of GPT-5, GPT-4o, GPT-4o-mini, Neva-22B, Gemini 1.5 Pro, and Gemini 2.5 Pro on image-based questions from the 66th to 69th National Examinations for Clinical Laboratory Technicians. The x-axis indicates the examination (66th to 69th), pooling the morning and afternoon sessions, and the y-axis represents accuracy. Blue bars denote the image-provided condition (image given), and orange bars represent the no-image condition (no image given). The red dashed line indicates the passing threshold of 60%. This figure enables comparison not only of performance differences among models but also of the impact of image availability on accuracy for image-based questions. Points denote the 3 per-run accuracies; error bars are 95% Wilson score CIs.

Figures 3 and 4 present a comparison of answer accuracies for LVLMs on all questions (text- and image-based) and on image-based questions, respectively, under 2 conditions: image-provided (image given) and no-image (no image given). In this section, we describe in detail how the presence of visual information influences model performance across different models and sessions.

On all questions, GPT-5 consistently outperformed the other models, achieving around 90.0% accuracy across all sessions, with the image-provided condition always surpassing the no-image condition. In particular, in the afternoon session of the 68th examination, GPT-5 achieved 97.0% with images and 84.7% without images, representing the highest performance among all models.

GPT-4o generally performed better with images, with accuracies ranging from 75.0% to 87.0% with images and 74.0% to 86.0% without images. Although some exceptions were observed, overall, image input improved its performance. GPT-4o-mini exhibited lower overall accuracy compared with other models, but in many sessions, performance was higher under the image-provided condition (46.3%‐60.7%) than under the no-image condition (45.3%‐57.3%).

Gemini 2.5 Pro also showed consistently higher accuracy under the image-provided condition in every session, such as 93.0% with images and 88.0% without images in the morning session of the 67th examination. Gemini 1.5 Pro showed accuracies ranging from 64.0% to 75.7% with images and 61.3% to 77.0% without. While in some sessions the no-image condition outperformed the with-image one, the overall differences were not substantial.

Neva-22B demonstrated the opposite trend, achieving accuracies of 25.0%‐37.0% with images and 28.0%‐39.0% without, indicating better performance without visual information. Moreover, frequent formatting errors were observed, such as blank responses or outputs that did not conform to the specified format.

**Figure 5.**
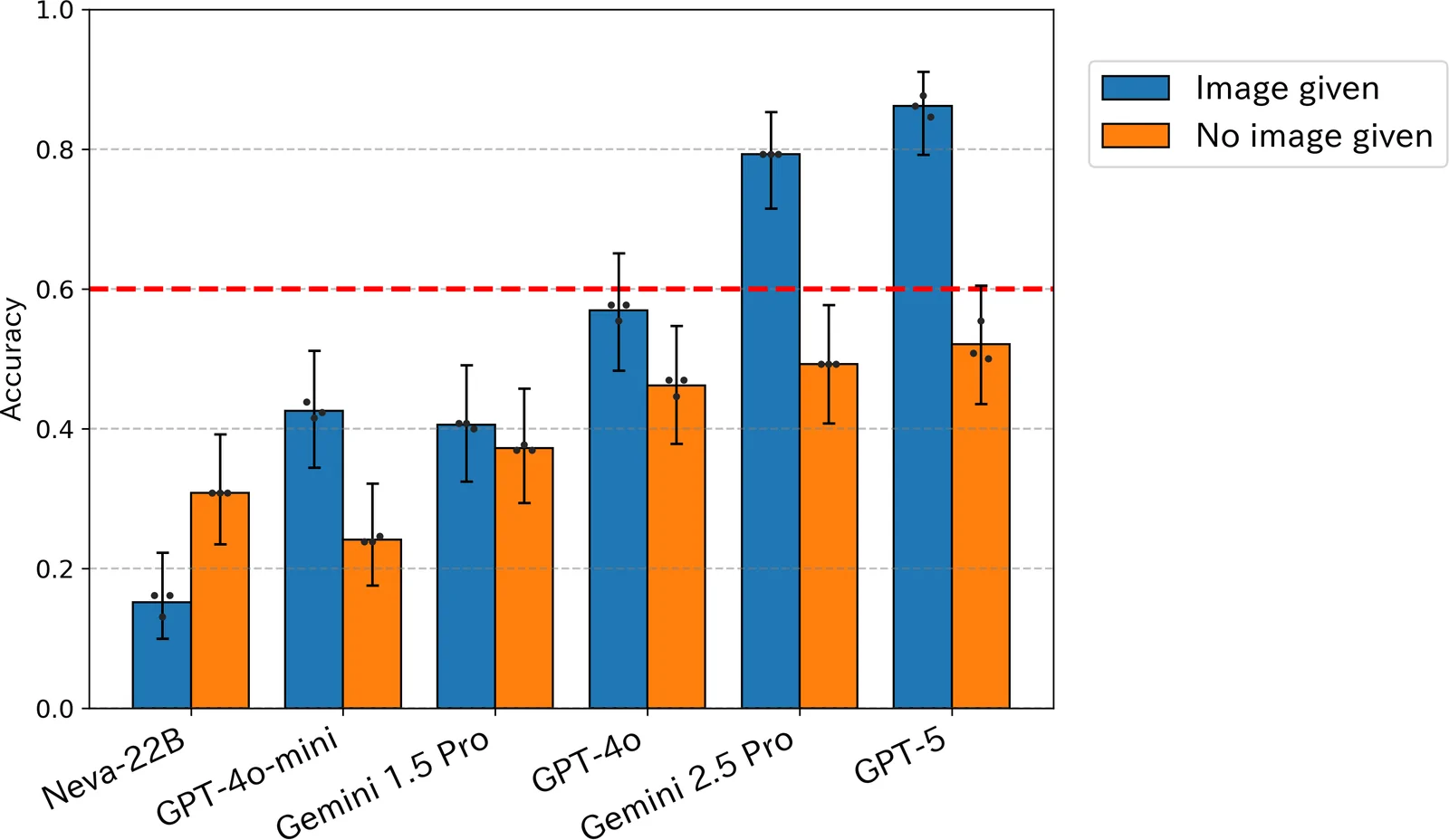
Comparison of average accuracy on image-based questions (n=130) for 6 LVLMs under the image-provided (image given) and no-image (no image given) conditions, pooled across the 66th to 69th National Examinations for Clinical Laboratory Technicians. The x-axis indicates the model, and the y-axis represents accuracy. Blue bars denote the image-given condition, and orange bars represent the no-image-given condition. Image input improved accuracy for all models except Neva-22B. The red dashed line represents the passing threshold of 60%. Points denote the 3 per-run accuracies; error bars are 95% Wilson score CIs.

In addition to the session-wise comparisons, we quantified the overall effect of image input using all image-based questions in Kensagishi QA (n=130), pooled across the 66th to 69th examinations (Figure 5). For each model, we compared accuracy under the image-provided condition (image given) against the no-image condition (no image given) and computed the absolute difference (ΔAcc=image given−no image given). Across the top-performing models, image input consistently improved accuracy: GPT-5 achieved 86.15% with images versus 52.05% without images (ΔAcc=34.10 points), and Gemini 2.5 Pro achieved 79.23% versus 49.23% (ΔAcc=30.00 points). GPT-4o also benefited from image input, with 56.92% versus 46.15% (ΔAcc=10.77 points). GPT-4o-mini improved from 24.10% to 42.56% (ΔAcc=18.46 points), while Gemini 1.5 Pro showed an increase from 37.18% to 40.51% (ΔAcc=3.33 points). In contrast, Neva-22B exhibited a negative image effect, decreasing from 30.77% (no image given) to 15.13% (image given; ΔAcc=–15.64 points). These results indicate that the impact of image input varies substantially across models.

### Modality-Specific Analysis of Image-Based Questions

To further investigate the sources of error in image-based questions, we categorized all 130 image-based questions into 5 visual modalities: (1) photographs (eg, histology images and instrument photographs; n=91), (2) graphs (n=21), (3) schematic diagrams (n=12), (4) tables (n=5), and (5) combined table+photograph items (n=1). As shown in Table 3, clear modality-dependent differences were observed. Photograph-based questions showed substantial performance gaps across models. For example, GPT-5 achieved 84.62% accuracy on photograph-based questions, Gemini 2.5 Pro 80.22%, and GPT-4o 59.34%, whereas Neva-22B achieved only 17.95%. In contrast, schematic diagrams yielded relatively high accuracy for top-performing models (Gemini 2.5 Pro: 91.67% and GPT-5: 97.22%), suggesting that structured visual abstractions were more robustly interpreted than naturalistic medical photographs. Graph-based questions were handled well by GPT-5 (82.54%), but showed lower accuracy for Gemini 2.5 Pro (61.90%) and substantial degradation in lower-performing models. Table-based questions demonstrated large intermodel variance, with Gemini 2.5 Pro and GPT-5 achieving 100.00% accuracy, and Gemini 1.5 Pro and Neva-22B achieving 20.00%.

**Table 3.** Accuracy by model and image modality, presented as percentages. For each model and image condition, accuracy is the mean across 3 independent runs (see Methods). The 130 image-based questions were grouped into 5 visual modalities; the n given in each column header is the number of image-based questions assigned to that modality, not a number of correct answers.

Model	Photograph (n=91), %	Graph (n=21), %	Diagram (n=12), %	Table (n=5), %	Table+photograph (n=1), %
GPT-4o-mini					
With image	47.62	23.81	41.67	40.00	0.00
Without image	27.11	15.87	27.78	0.00	0.00
GPT-4o					
With image	59.34	46.03	52.78	60.00	100.00
Without image	52.01	20.63	44.44	40.00	100.00
Gemini 1.5 Pro					
With image	44.69	38.10	25.00	20.00	0.00
Without image	43.96	20.63	16.67	40.00	0.00
Gemini 2.5 Pro					
With image	80.22	61.90	91.67	100.00	100.00
Without image	52.75	38.10	58.33	20.00	0.00
Neva-22B					
With image	17.95	3.17	13.89	20.00	0.00
Without image	35.16	19.05	25.00	20.00	0.00
GPT-5					
With image	84.62	82.54	97.22	100.00	100.00
Without image	56.04	46.03	30.56	46.67	100.00

### Model Accuracy Across Medical Subfields

**Figure 6.**
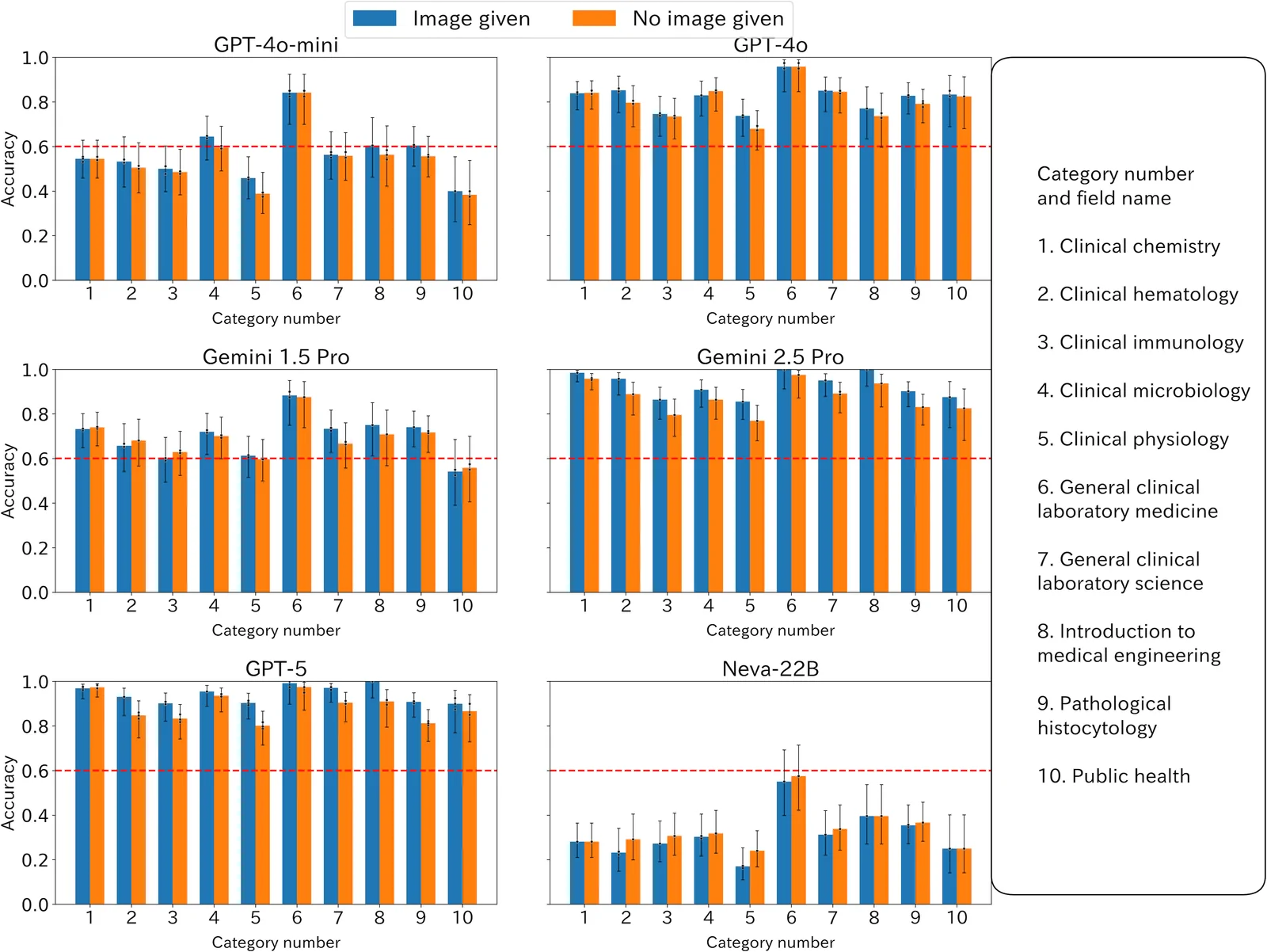
Accuracy by medical subfield for each model—GPT-5, GPT-4o, GPT-4o-mini, Neva-22B, Gemini 1.5 Pro, and Gemini 2.5 Pro—on all questions (both text- and image-based) from the 66th to 69th National Examinations for Clinical Laboratory Technicians. The x-axis represents medical subfields, and the y-axis represents accuracy. Blue bars indicate accuracy under the image-given condition (where images were provided for all questions), while orange bars represent the no-image-given condition (no images provided, including for image-based questions). The red dashed line indicates the passing threshold of 60%. For each model, points denote the 3 per-run accuracies, and error bars are 95% Wilson score CIs.

To assess how model performance varies across different areas of medical knowledge, we aggregated the accuracy of each LVLM by medical subfield (Figure 6). The 10 targeted subfields were clinical chemistry, clinical hematology, clinical immunology, clinical microbiology, clinical physiology, general clinical laboratory medicine, general clinical laboratory science, introduction to medical engineering, pathological histocytology, and public health.

GPT-5 achieved exceptionally high performance across all subfields, with accuracies ranging from approximately 90.0% to 100.0%. In the introduction to medical engineering, it reached 100.0%, while in general clinical laboratory medicine, it achieved 99.2% under the image-given condition and 97.5% under the no-image-given condition. Similarly, in clinical chemistry and clinical microbiology, GPT-5 maintained stable accuracy around 95.0% regardless of image availability. Although its performance declined slightly in the no-image-given condition for clinical physiology (80.1%) and pathological histocytology (81.3%), accuracy remained above 80.0% in all subfields.

Gemini 2.5 Pro demonstrated consistently high accuracy across most subfields, typically in the 80.0%‐90.0% range, and achieved 100.0% accuracy in both general clinical laboratory medicine and introduction to medical engineering. GPT-4o also showed robust performance, with accuracy generally in the 70.0%‐80.0% range and exceeding 90.0% in general clinical laboratory medicine.

In contrast, Gemini 1.5 Pro exhibited substantial variability across subfields. Although some sessions in general clinical laboratory medicine reached 100.0% accuracy, performance in clinical physiology and public health fell below 50.0% in several sessions. GPT-4o-mini and Neva-22B showed lower overall accuracy across subfields, with Neva-22B remaining below 50.0% accuracy in all subfields.

To contextualize differences in subfield-level performance, we quantified the proportion of image-based questions within each subfield and classified them into visual-heavy and text-heavy subfields. Clinical physiology (37/104, 35.6%), clinical hematology (24/72, 33.3%), pathological histocytology (23/112, 20.5%), clinical immunology (14/88, 15.9%), and general clinical laboratory science (12/80, 15.0%) were categorized as visual-heavy subfields. In contrast, clinical microbiology, introduction to medical engineering, public health, general clinical laboratory medicine, and clinical chemistry contained relatively few image-based questions and were categorized as text-heavy.

**Figure 7.**
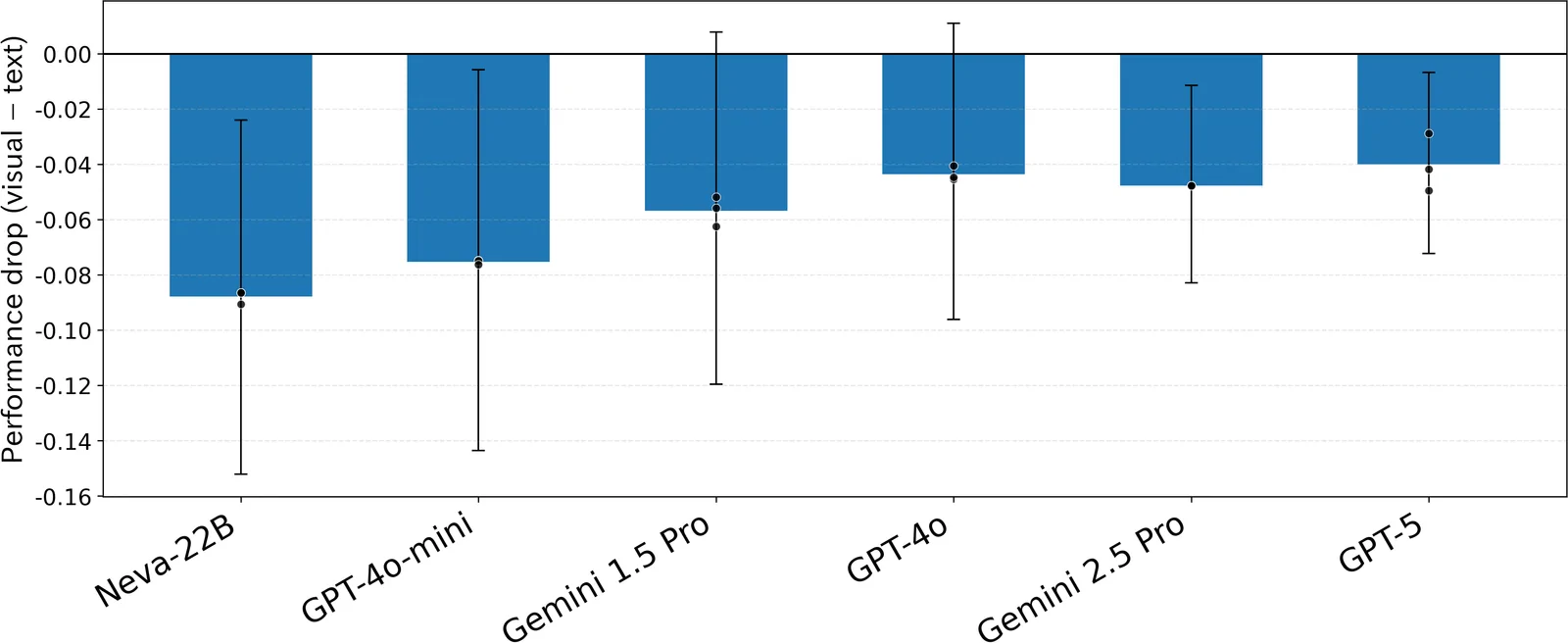
Effect size of visual-heavy subfields under the image-given condition, defined as the difference in accuracy between visual-heavy and text-heavy subfields (visual-heavy minus text-heavy). Negative values indicate lower accuracy in visual-heavy domains. All models exhibited a performance drop in visual-heavy subfields, with larger drops observed for GPT-4o-mini and Neva-22B and smaller drops for GPT-5 and Gemini 2.5 Pro, indicating greater robustness of higher-capacity models to visually demanding content. Points denote the 3 per-run effect sizes; the error bar is the 95% Newcombe hybrid-score CI for the visual-minus-text difference.

Grouping subfields into visual-heavy and text-heavy categories revealed a consistent trend across models (Figure 7). All models demonstrated lower accuracy in visual-heavy subfields compared with text-heavy subfields, as indicated by negative effect sizes (visual-heavy minus text-heavy). The magnitude of this performance gap varied by model capacity: GPT-5 and Gemini 2.5 Pro exhibited relatively small differences, indicating robustness to visually demanding content, whereas GPT-4o-mini and Neva-22B showed substantially larger negative effect sizes. These findings suggest that visually intensive disciplines pose greater challenges for lower-capacity models and that higher-capacity models are more resilient to the increased cognitive and multimodal demands of visual-heavy medical subfields.

### Correlation With Human Performance

**Figure 8.**
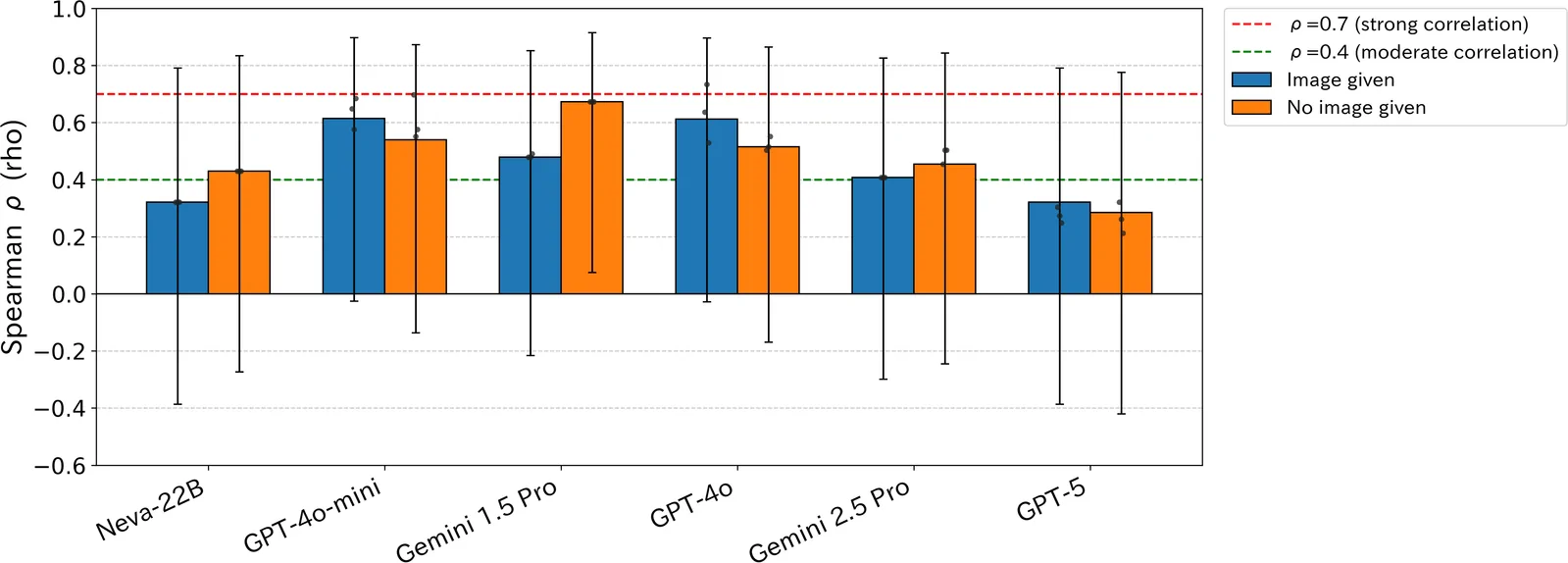
Comparison of Spearman rank correlation coefficients (ρ) for each model under image-provided (image given) and no-image (no image given) conditions, with exact *P* values reported in the main text. Based on human and model accuracy, medical subfields were ranked, and Spearman rank correlation was calculated. The red dashed line indicates a reference value for strong correlation (ρ=0.7), while the green dashed line indicates a reference value for moderate correlation (ρ=0.4). This figure quantitatively illustrates how closely each model’s performance aligns with human subfield-level scoring trends. Points denote the Spearman coefficient recomputed from each of the 3 individual runs, and error bars are 95% Fisher z CIs (n=10 subfields).

To evaluate the consistency between model outputs and human accuracy rankings by subfield, we calculated the Spearman rank correlation coefficient (Figure 8). This metric indicates how closely each model mirrors the domain-specific performance trends observed in human examinees; a coefficient closer to 1 implies stronger alignment with human rankings.

GPT-4o showed relatively high correlations, with ρ=0.61 (95% CI –0.03 to 0.90; *P*=.06) under the image-provided condition and ρ=0.52 (95% CI –0.17 to 0.86; *P*=.13) under the no-image condition, though neither reached statistical significance. GPT-4o-mini exhibited similar results, achieving ρ=0.61 (95% CI –0.03 to 0.90; *P*=.06) with images and ρ=0.54 (95% CI –0.14 to 0.87; *P*=.11) without images, with the image-provided condition approaching significance.

GPT-5 showed ρ=0.32 (95% CI –0.39 to 0.79; *P*=.37) under the image-provided condition, and ρ=0.28 (95% CI –0.42 to 0.78; *P*=.43) under the no-image condition. These weaker correlations indicate limited consistency with human rankings.

Gemini 1.5 Pro recorded the highest correlation under the no-image condition, with ρ=0.67 (95% CI 0.07‐0.91; *P*=.03), which reached statistical significance. In contrast, under the image-provided condition it showed a lower correlation (ρ=0.48, 95% CI –0.22 to 0.85; *P*=.16), not reaching significance.

Gemini 2.5 Pro showed moderate correlations—ρ=0.45 (95% CI –0.25 to 0.84; *P*=.19) without images and ρ=0.41 (95% CI –0.30 to 0.83; *P*=.24) with images—but neither was significant. Neva-22B exhibited low correlations in both conditions (ρ=0.43, 95% CI –0.27 to 0.83; *P*=.21 without images; ρ=0.32, 95% CI –0.39 to 0.79; *P*=.37 with images), suggesting little alignment with human rankings.

### Evaluation of Model Performance on Unseen Questions After Knowledge Cutoff

All examination questions used in prior evaluations were published before the knowledge cutoff dates of the respective models, raising the possibility that the models may have directly encountered these questions or answer choices during training. To address this concern, we evaluated model performance using the 71st National Examination for Clinical Laboratory Technicians (administered in 2025), which consists of 200 newly released questions (100 in the morning and 100 in the afternoon) published after the respective models’ knowledge cutoffs.

**Figure 9.**
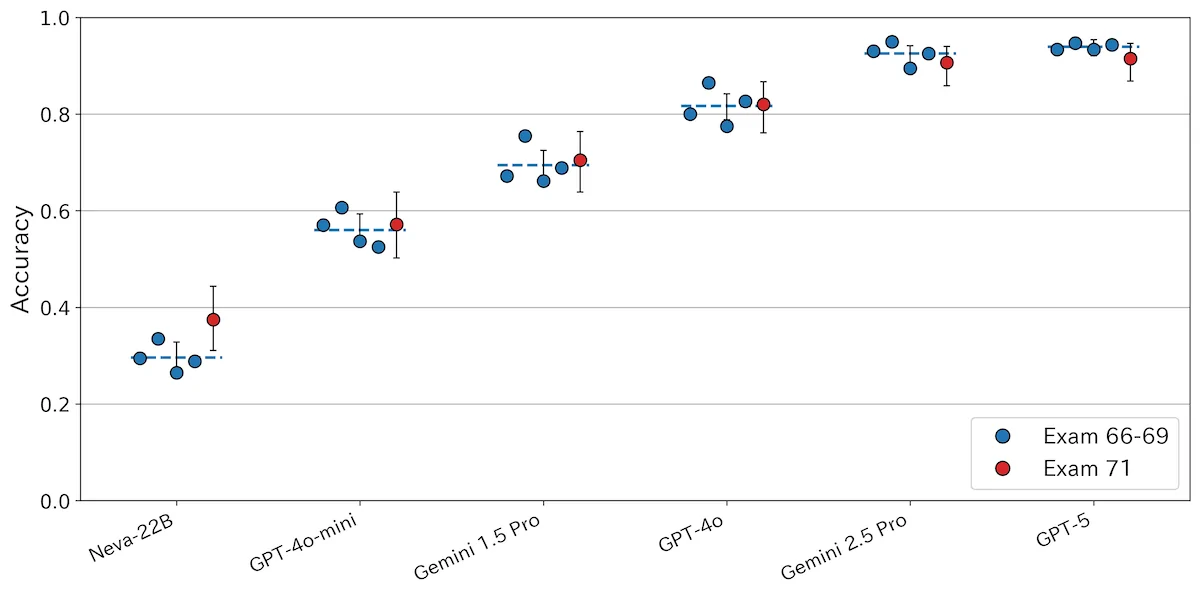
Comparison of model accuracy before and after the knowledge cutoff. The figure shows, for each model—GPT-5, GPT-4o, GPT-4o-mini, Neva-22B, Gemini 1.5 Pro, and Gemini 2.5 Pro—the average accuracy from 2020‐2023 (blue) and the accuracy on the 71st National Examination for Clinical Laboratory Technicians released in 2025 (red), which was published after the knowledge cutoff. Across all models, the 2025 accuracy was comparable to or higher than the historical average, with no significant performance degradation observed. The blue dashed line indicates the average accuracy of each model across the 66th to 69th examinations. Blue points are the 4 yearly accuracies; error bars are 95% Wilson score CIs for the 2020‐2023 mean (n=800) and for 2025 (n=200).

As a result, GPT-5 achieved 91.5%, Gemini 2.5 Pro 90.7%, GPT-4o 82.0%, Gemini 1.5 Pro 70.5%, GPT-4o-mini 57.2%, and Neva-22B 37.5% (Figure 9). None of the models exhibited substantial deviations from their respective average accuracies over the previous 4 years (2020‐2023). The largest increase was observed in Neva-22B, which improved by 7.9 points compared with its historical average of 29.6%. GPT-4o-mini also showed a modest increase of 1.2 points relative to its past average of 56.0%. By contrast, GPT-5 slightly declined from 93.9% to 91.5% (–2.4 points), and Gemini 2.5 Pro from 92.5% to 90.7% (–1.8 points).

To statistically validate these observations, we performed chi-square tests comparing the accuracy between the training years (2020‐2023) and the 2025 examination for each model and image condition. As summarized in Table 4, none of the comparisons showed statistically significant declines (*P*≥.05). These results confirm that the observed declines in accuracy are not statistically significant and support the conclusion that no material performance degradation was observed in the 2025 examination. These findings indicate that all models maintained comparable accuracy when applied to previously unseen questions created after their respective knowledge cutoffs, with no significant performance degradation observed.

**Table 4.** Chi-square test results comparing model accuracy between the training years (2020‐2023; n=800 questions) and the 2025 examination (n=200 questions). For each model and image condition, accuracy is the mean across 3 independent runs (see Methods). A 2×2 contingency table of correct versus incorrect responses was constructed using question-level counts. ΔAcc denotes the difference in accuracy (2025 minus 2020‐2023, in percentage points), with its two-sided 95% CI computed by the Newcombe hybrid score method.

Model and condition		2020‐2023 Acc (%)	2025 Acc (%)	ΔAcc (pp)^a^	95% CI of ΔAcc (pp)	*P* value
GPT-4o-mini						
Image given		55.96	57.17	1.21	–6.51 to 8.71	.80
No image given		53.04	55.67	2.62	–5.11 to 10.18	.53
GPT-4o						
Image given		81.67	82.00	0.33	–6.10 to 5.82	.90
No image given		79.75	79.50	–0.25	–6.92 to 5.55	.94
Gemini 1.5 Pro						
Image given		69.42	70.50	1.08	–6.26 to 7.82	.76
No image given		68.46	67.83	–0.62	–8.07 to 6.30	.89
Gemini 2.5 Pro						
Image given		92.50	90.67	–1.83	–6.93 to 2.04	.35
No image given		86.75	87.67	0.92	–4.79 to 5.53	.78
Neva-22B						
Image given		29.58	37.50	7.92	0.72 to 15.45	.03
No image given		32.12	38.50	6.38	–0.89 to 13.96	.09
GPT-5						
Image given		93.92	91.50	–2.42	–7.33 to 1.23	.23
No image given		88.17	86.50	–1.67	–7.47 to 3.06	.53

a pp: percentage point.

## Discussion

### Principal Findings

This study systematically evaluated multiple LVLMs using Kensagishi QA, an examination-specific benchmark constructed from the Japanese National Examination for Clinical Laboratory Technicians (2020‐2023), and demonstrated that several recent cloud-hosted models exceeded the human passing threshold. At the same time, substantial differences were observed across models in overall accuracy, visual integration, and alignment with human performance patterns, clarifying their respective performance characteristics and applicability in medical contexts.

### Interpretation and Comparison With Prior Work

At a high level, recent cloud-hosted LVLMs exceeded the human passing threshold, with GPT-5 showing the strongest overall performance, Gemini 2.5 Pro close behind, and GPT-4o also performing robustly. Gemini 1.5 Pro exhibited greater variability by subfield and condition, whereas GPT-4o-mini and Neva-22B performed relatively poorly overall. Crucially, for image-based questions, providing images improved accuracy for the top-performing models (eg, GPT-5, Gemini 2.5 Pro, and GPT-4o), indicating that visual input contributes materially to performance; by contrast, Neva-22B did not show such gains. These findings suggest that the ability to integrate visual information is a key driver of accuracy in this examination setting. Importantly, this effect was not universal but model-dependent, underscoring that multimodal capability cannot be assumed even when visual input is available.

These findings are broadly consistent with previous studies showing that recent LLMs can achieve high performance on medical licensing examinations. Med-PaLM 2 demonstrated expert-level performance on MedQA and other medical question-answering benchmarks, while subsequent studies incorporating structured clinical knowledge further improved performance on USMLE-style examinations [8,10]. In the Japanese context, GPT-4 successfully passed multiple years of the Japanese National Medical Licensing Examination using the text-based IgakuQA benchmark [11]. Previous work has also reported that GPT-4-class models achieve passing-level performance on the Japanese National Examination for Clinical Laboratory Technicians [13]. Our results are consistent with these reports but extend them in 3 important respects: we evaluated multiple model generations and providers under a unified protocol, explicitly quantified the contribution of visual input using paired image-provided and no-image conditions, and additionally evaluated questions released after the reported knowledge cutoffs.

The paired image-provided and no-image evaluation also provides insights that cannot be obtained from conventional text-only examination benchmarks. Previous multimodal medical AI studies have demonstrated that vision-language models can answer biomedical image questions [26,27]; however, relatively little has been reported regarding the extent to which visual input contributes to performance on professional licensing examinations. In our benchmark, image provision substantially improved performance for GPT-5, Gemini 2.5 Pro, and GPT-4o, whereas Neva-22B showed no benefit. These findings indicate that multimodal capability should not be regarded as a binary property. Instead, the effectiveness of visual information integration differs markedly among models, even when all models technically accept image input.

Subfield-level patterns further suggest that performance reflects not only overall model capacity but also the cognitive demands of each subfield. For example, the relatively strong performance of smaller models in general clinical laboratory science may reflect its emphasis on standardized procedures and conceptual knowledge, whereas visually intensive or quantitatively demanding subfields (eg, pathological histocytology and clinical chemistry) showed larger gaps between high- and lower-capacity models.

Beyond absolute accuracy, we examined whether models mirrored human domain-level patterns. GPT-4o (and, to a lesser extent, GPT-4o-mini) displayed relatively strong rank alignment with human subfield trends, while GPT-5 achieved the highest accuracy with more limited alignment. Gemini 1.5 Pro showed the strongest human alignment when images were withheld but weaker alignment with images provided. This divergence suggests potentially different use cases; however, strong alignment with human difficulty patterns does not necessarily imply superior pedagogical utility, as it may also reflect shared error tendencies rather than improved explanatory capacity.

To probe generalizability beyond potential training-set exposure, we evaluated all models on the 71st examination (administered in 2025), released after each model’s knowledge cutoff. Accuracies were comparable to historical averages from 2020‐2023, indicating no substantial degradation on unseen questions. This suggests that the evaluated LVLMs leverage transferable knowledge and reasoning rather than memorization of specific items and that the examination’s structure and content remain sufficiently stable for such capabilities to carry over.

For practical deployment, the implications of the present findings are primarily educational rather than clinical. Kensagishi QA provides a standardized benchmark for comparing cloud-hosted LVLMs before their adoption in examination preparation systems or educational support tools. The marked effect of visual input also suggests that evaluations intended for visually intensive subjects should include the original examination images rather than relying on text-only transcriptions. At the same time, our study evaluated only answer selection and did not assess explanation quality, hallucinations, calibration, or educational effectiveness [28]. Therefore, high examination accuracy alone should not be interpreted as sufficient evidence for deployment in either educational or clinical settings without additional validation.

### Limitations

Several limitations should be considered. First, our evaluation focused exclusively on multiple-choice accuracy and did not assess reliability-related dimensions such as factuality, instruction following, safety [28-30], or appropriate handling of Japanese medical terminology [11,31]. Second, all experiments were conducted under a single “numbers-only” direct-answer prompting condition, which may not fully elicit multi-step reasoning. Prior work has reported that reasoning-oriented prompting strategies, including chain-of-thought, can improve performance in some medical and complex reasoning tasks [8,9,32]. Therefore, the reported accuracies should be interpreted as standardized baseline performance under controlled prompting conditions rather than prompt-optimized upper-bound estimates. Third, the number of image-based questions (n=130) was relatively small, limiting statistical precision in estimating multimodal effects and model-by-image interactions. Fourth, our evaluation did not include high-capacity or medically specialized open-weight LVLMs [26,27]; thus, the present results should not be interpreted as a direct comparison between open- and closed-weight models.

### Conclusions

Looking ahead, we plan to broaden coverage to additional model families (including high-capacity and medically specialized open-weight, self-hosted medical LVLMs) under a common protocol; incorporate multidimensional quality metrics that combine accuracy with factuality, instruction compliance, safety, and terminology handling [28,29]; refine prompt design to better leverage domain knowledge [33]; and investigate the interplay between retrieval-augmented approaches and intrinsic reasoning for tasks such as differential diagnosis. Systematic comparison between cloud-hosted and open-weight LVLMs will be necessary to determine whether observed performance differences reflect deployment modality, model scale, architectural design, or other factors. Ultimately, these efforts will inform the development of learning-support tools that visualize mastery, target weak areas, and provide explanatory feedback for national examination preparation and broader medical education [34]. In summary, by providing the first systematic, multi-model evaluation of cloud-hosted LVLMs on this examination with explicit isolation of visual input—extending prior text-only, few-model studies—this work delivers a reproducible Kensagishi QA benchmark and actionable, use-case–specific guidance for deploying LVLMs in medical education and clinical training. More broadly, this framework illustrates how nationally specific professional examinations can serve as rigorous, multilingual testbeds for evaluating and improving medical AI systems in realistic, high-stakes contexts.

## Abbreviations

GPU: graphics processing unit
Kensagishi QA: Kensagishi question-answer
LLM: large language model
LVLM: large vision-language model
MHLW: Ministry of Health, Labour and Welfare
PDL1.0: Public Data License version 1.0
SCAI: Semantic Clinical AI
USMLE: United States Medical Licensing Examination
